# A Test of Evolutionary Policing Theory with Data from Human Societies

**DOI:** 10.1371/journal.pone.0024350

**Published:** 2011-09-01

**Authors:** Rolf Kümmerli

**Affiliations:** 1 Department of Environmental Microbiology, Swiss Federal Institute of Aquatic Science and Technology (Eawag), Dübendorf, Switzerland; 2 Department of Environmental Sciences, Swiss Federal Institute of Technology Zurich, Zürich, Switzerland; University of Maribor, Slovenia

## Abstract

In social groups where relatedness among interacting individuals is low, cooperation can often only be maintained through mechanisms that repress competition among group members. Repression-of-competition mechanisms, such as policing and punishment, seem to be of particular importance in human societies, where cooperative interactions often occur among unrelated individuals. In line with this view, economic games have shown that the ability to punish defectors enforces cooperation among humans. Here, I examine a real-world example of a repression-of-competition system, the police institutions common to modern human societies. Specifically, I test evolutionary policing theory by comparing data on policing effort, per capita crime rate, and similarity (used as a proxy for genetic relatedness) among citizens across the 26 cantons of Switzerland. This comparison revealed full support for all three predictions of evolutionary policing theory. First, when controlling for policing efforts, crime rate correlated negatively with the similarity among citizens. This is in line with the prediction that high similarity results in higher levels of cooperative self-restraint (i.e. lower crime rates) because it aligns the interests of individuals. Second, policing effort correlated negatively with the similarity among citizens, supporting the prediction that more policing is required to enforce cooperation in low-similarity societies, where individuals' interests diverge most. Third, increased policing efforts were associated with reductions in crime rates, indicating that policing indeed enforces cooperation. These analyses strongly indicate that humans respond to cues of their social environment and adjust cheating and policing behaviour as predicted by evolutionary policing theory.

## Introduction

Ever since Darwin [1], cooperative behaviours have puzzled evolutionary biologists, as it is difficult to understand why natural selection should favour traits that benefit other individuals. Inclusive fitness theory [2] provides a solution to that problem by showing that a cooperative trait can be selected for when the fitness cost (*c*) to the actor is smaller than the fitness benefit (*b*) to the recipient times the relatedness (*r*) between the two: *rb*>*c* (Hamilton's rule). Accordingly, Hamilton's rule can be satisfied when cooperation provides direct fitness benefits (i.e. mutual beneficial cooperation with *c*<0), or when cooperation provides indirect (kin-selected) fitness benefits (i.e. altruistic cooperation with *c*>0) [3]–[5]. For both types of cooperative behaviours, low relatedness introduces divergence in reproductive interests among interacting individuals, thereby promoting selfish behaviours [6]. Consequently, under low-relatedness conditions cooperation can often only be maintained through mechanisms that repress competition among group members. Repression of competition mechanisms, such as policing [7]–[10], punishment [11], [12], sanctions [13], [14], and randomization of reproductive success [15], [16], enforce cooperation because they unite the proximate interests of group members, such that individuals can only increase their inclusive fitness by maximizing the reproductive output of the group [16]–[23]. For example, social insect workers in low-relatedness societies often police their co-workers by destroying their selfishly laid eggs, thereby potentially maximizing colony productivity and guaranteeing a fair share of indirect fitness benefits among colony members [24]. Similarly, studies have shown that the possibility to punish non-cooperative individuals enforces cooperation among unrelated humans in economic games [25], [26], thereby guaranteeing a fair share of direct benefits of cooperation.

Despite the awareness that repression-of-competition mechanisms seem to be of central importance in human societies [17], [18], [25]–[27], real-world systems such as the sophisticated policing institutions common to modern human societies have virtually attracted no attention by evolutionary biologists. This contrasts with the long-standing interest among economists to understand economic aspects of policing [28]–[32]. Consequently, general information on the behavioural ecology of human policing is lacking and it is unknown whether humans respond to changes in policing efforts and community demography as predicted by evolutionary policing theory [20], [21], [33].

Here, I conduct a test of evolutionary policing theory by comparing policing data across the 26 cantons of Switzerland. The cantons of Switzerland provide a unique and highly suitable system for such a test because each canton represents a politically independent republic that features an independent policing system, which at the same time must adhere to the federal code of law. Because data collection on policing and many other demographic variables is coordinated at the federal level and is based on standardized protocols, data are readily comparable among cantons.

For each canton, I extracted relevant data from the Swiss Statistical Encyclopedia (SSE) – an open-access database – to test the three main predictions of evolutionary policing theory [20]. The first prediction holds that in the absence of policing, increased relatedness among members of a social group leads to higher levels of cooperative self-restraint (i.e. lower levels of defection). In other words, high relatedness aligns the interests of individuals, which is predicted to result in higher levels of cooperation. The second prediction holds that the policing effort is a negative function of relatedness. Put simply, more policing is required to enforce cooperation under low-relatedness conditions, where interests among individuals diverge most. The third prediction holds that higher policing efforts enforce cooperation more efficiently thereby resulting in higher levels of cooperation. Three variables are needed to test these predictions, which are: (i) the level of cooperation/defection; (ii) policing effort; and (iii) relatedness among interacting individuals. In economic games, the level of cooperation or defection is usually given by a subject's respective decision to contribute or not to contribute monetary units to a public good [34]. Such individual-based levels of cooperation and defection cannot be obtained from comparative data sets as used here. However, in the current context cooperation can be regarded as an act of self-restraint, whereby cooperative individuals are the ones that obey the law, whereas defecting individuals are the ones that violate the law. Consequently, the per capita crime rate can be regarded as a proxy for the level of defection, and an inverse proxy for the level of cooperative self-restraint at the community level (see [9], [32] for using similar approaches). To estimate policing effort, I calculated the per capita monetary investment into policing. To obtain a proxy for relatedness among citizens, I defined a similarity index, which combined data on community size (i.e. number of citizens) and proportion of foreigners. The reasoning here is that humans, although today mostly living in societies where relatedness is low, have likely evolved the ability to respond to cues of relatedness in the past, when cooperative interactions occurred in much smaller societies and probably preferentially among related individuals [35]. It is likely that people have retained the ability to respond to these cues, irrespective of the current adaptive consequences. This is reflected by laboratory studies, showing that humans respond to cues of increased similarity by up-regulating cooperation [36]–[38].

## Methods

### Data collection

I obtained data on crime rates, monetary investment into policing, the number of citizens, and proportion of foreigners from SSE (http://www.bfs.admin.ch/bfs/portal/en/index/infothek/lexikon.html) – an open-access database provided by the Swiss government (Table 1). Consequently, this study is based on a comparative approach, and does not involve human participants. Therefore, no approval by the author's institutional ethical review board was needed for this study.

**Table 1:** Population demography, policing expenses and registered crimes of the 26 cantons of Switzerland.

Canton	Number of citizens in thousands*	Percentage of foreigners*	Policing expenses in million CHF^+^	Number of registered crimes*
Aargau	600.0	21.5	156.1	32735
Appenzell Innerrhoden	15.7	10.0	3.8	419
Appenzell Ausserrhoden	53.0	13.9	13.2	2367
Bern	974.2	13.0	368.7	67800
Basel-Landschaft	272.8	18.9	78.6	13962
Basel-Stadt	187.9	31.5	145.1	20467
Fribourg	273.2	17.7	83.9	14391
Genève	453.3	38.7	360.1	63905
Glarus	38.5	19.8	20.7	1532
Graubünden	191.9	16.1	91.9	8156
Jura	70.1	12.3	22.0	2986
Luzern	373.0	16.4	112.7	23229
Neuchâtel	171.6	23.1	70.2	13429
Nidwalden	40.8	10.7	9.1	1287
Obwalden	35.0	12.9	8.1	1504
St. Gallen	474.7	21.7	128.4	24162
Schaffhausen	75.7	22.9	32.0	4296
Solothurn	252.7	19.3	89.3	16216
Schwyz	144.7	18.0	41.0	5370
Thurgau	244.8	21.0	55.7	11347
Ticino	335.7	25.4	129.1	20236
Uri	35.3	9.4	21.4	1069
Vaud	701.5	30.5	283.2	58467
Valais	307.4	20.4	102.9	15114
Zug	110.9	23.3	46.5	7264
Zürich	1351.3	23.7	851.0	117099

*data from 2009 / + data from 2008.

For the per capita crime rate, I considered crimes that violated the main code of law (i.e. the ‘Schweizerische Strafgesetzbuch’, StGB) and divided the number of registered crimes by the number of citizens. The StGB covers all types of crimes, except crimes related to drug abuse/dealing and violation of traffic rules (i.e. 82% of all crimes reported in Switzerland in 2009 fall under the StGB). For the policing effort, I divided the amount of tax money invested into policing by the number of citizens. To obtain a proxy for relatedness, I calculated a similarity index (*s*) as follows. I first defined dissimilarity (*d*) among citizens as *d*  =  *w*log(*c*) + *f*, where log(*c*) is the natural logarithm of the number of citizens, *f* is the proportion of foreigners, and *w* is a scaling factor such that both addends are weighted equally. I then calculated *s*  = 1-*d*/*d*_max_, where *d*_max_ represents the highest dissimilarity value observed among all cantons. Consequently, *s* ranges between zero and one, whereby *s* = 0 for the canton with *d*_max_.

I used data from 2009 for crime rates, the number of citizens, and the proportion foreigners, whereas for the monetary investment into policing, I used data from 2008, the most recent data set available. This was not a problem as cantonal investment into policing highly correlated between years (e.g. between 2007 and 2008: Pearson's product moment correlation *r*>0.999). I further repeated analyses with data sets from 2005 and 2007 to examine the generality of my findings. For these earlier years, I obtained data on number of citizens, proportion of foreigners and monetary investment into policing from SSE. For crime rates, I obtained data from the cantonal bureaus of statistics, because no standardized federal data sets were available for these earlier years.

### Statistical analysis

To test the first prediction of policing theory – increased relatedness leads to lower crime rates (i.e. higher levels of cooperation) in the absence of policing – I conducted a partial correlation analysis, where I examined the relationship between the similarity index and the per capita crime rate, whilst controlling for policing effort. To test the second prediction of policing theory – higher policing effort is required with lower relatedness – I used Pearson's product-moment correlation to examine the relationship between per capita monetary investment into policing and the similarity index. To test the third prediction of policing theory – higher policing effort reduces crime rate (i.e. increases the level of cooperation) – I first used Pearson's product-moment correlation to examine the relationship between per capita monetary investment into policing and the per capita crime rate. The test of this last prediction was the main focus of numerous economical studies, which yielded controversial results (reviewed in [30], [32]). The reason for this controversy was that in a specific year the policing effort is often the product of crime rates and not vice versa, which prevents testing the third prediction of evolutionary policing theory. To control for that problem, I conducted an alternative test of this prediction by relating between-year changes in policing efforts to between-year changes in crime rates. Here, one would predict that crime rates should decrease or increase in cantons that extend or reduce their policing efforts, respectively. For this analysis, I compared data from 2005 and 2007 (note that data from 2009 could not be used for such a comparison because a new standardized method for data collection was used from this year onwards). As the testing of these hypotheses involved multiple pairwise comparisons, I applied the false discovery rate control method [39] to adjust the nominal α = 0.05. All statistical analyses were conducted with R 2.11.1 (http://www.R-project.org).

## Results

**Figure 1:**
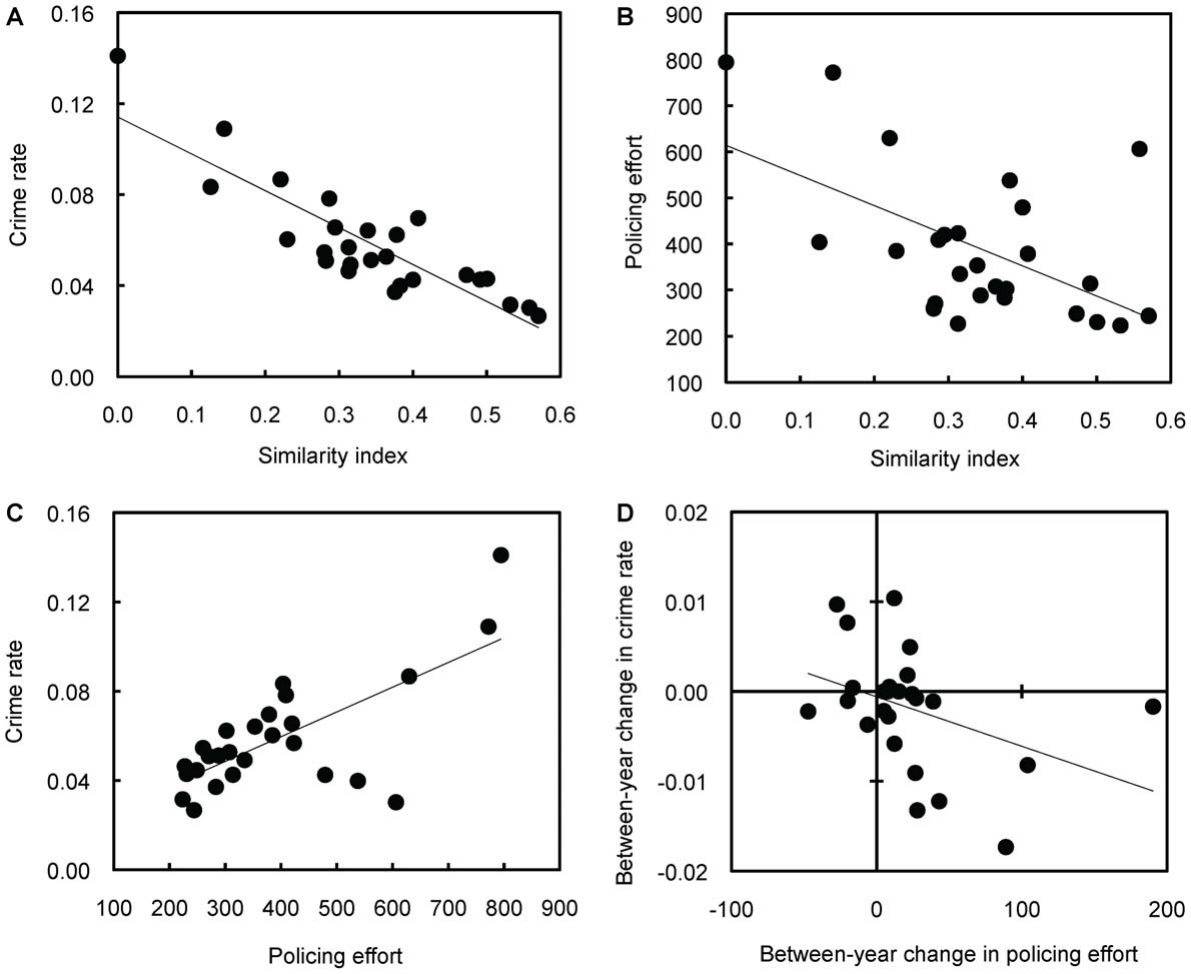
Testing predictions of evolutionary policing theory with data from human societies. Significant correlations (indicated by trend lines) between: (a) the per capita crime rate and the similarity index; (b) the policing effort (per capita investment into policing) and the similarity index; (c) the policing effort and the per capita crime rate; (d) the between-year change in policing effort and crime rate. Each data point represents one out of the 26 cantons of Switzerland.

I found strong support for the first and the second prediction of policing theory in all three study years (Table 2). First, when statistically controlling for policing efforts, per capita crime rates were significantly lower in societies with higher similarity indexes (Figure 1A). Second, policing efforts were significantly lower in societies with higher similarity values (Figure 1B).

**Table 2:** Predictions of evolutionary policing theory [20] and the corresponding empirical tests using data from the 26 cantons of Switzerland in 2005, 2007, and 2009.

Prediction	Year	Level of comparison	Correlation coefficient	*P*-value	Reference to figure
Negative correlation between crime rate and similarity index	2005	cantons	−0.561	0.0066	-
	2007	cantons	−0.714	<0.0001	-
	2009	cantons	−0.805	<0.0001	Figure 1A
Negative correlation between policing effort and similarity index	2005	cantons	−0.594	0.0014	-
	2007	cantons	−0.527	0.0057	-
	2009	cantons	−0.541	0.0043	Figure 1B
Negative correlation between policing effort and crime rate	2005	cantons	0.767	<0.0001	-
	2007	cantons	0.744	<0.0001	-
	2009	cantons	0.703	<0.0001	Figure 1C
	2005/2007	years	−0.440	0.0405	Figure 1D

In contrast, when relating policing efforts to per capita crime rates, there was first no support for the third prediction of policing theory (Table 2), as policing efforts correlated positively, and not negatively, with crime rates in all three study years (Figure 1C). This finding indicates towards the problem of causality well known from economic studies [30], [32], which showed that at any given moment in time the policing effort is dictated by the crime rate (i.e. higher crime rates demand disproportionally large policing efforts). Conversely, when I related yearly changes in policing efforts to yearly changes in crime rates, I found that cantons that increased policing efforts from 2005 to 2007 showed an average decrease in crime rates (*n* = 16; decrease in per capita crime rate  = −0.0036±0.0018), whereas cantons that reduced policing efforts between years showed an average increase in crime rates (*n* = 6; increase in per capita crime rate  = 0.0018±0.0023; one-tailed t-test between the two categories: *t*_20_ = −1.87, *P* = 0.038). Overall, there was a significant negative correlation between the yearly changes in policing efforts and crime rates (Table 2, Figure 1D), a finding that is fully compatible with the third prediction of evolutionary policing theory.

## Discussion

By relating demographic data to crime rates and policing efforts across the 26 cantons of Switzerland, I found full support of all three predictions of evolutionary policing theory [20]. Specifically, I show that: (i) when controlling for policing efforts, crime rates decreased with higher similarities among citizens; (ii) higher policing efforts were observed when similarity among citizens was low; (iii) increased policing efforts went along with a reduction in crime rates. These analyses strongly indicate that humans respond to cues of their social environment and adjust cheating and policing behaviour accordingly.

The first finding, showing that crime rates were lower in societies with high similarity indexes, suggests that similarity among citizens can be considered analogous to genetic relatedness as used in Hamilton's rule. Specifically, it seems that high similarity, analogous to high genetic relatedness, aligns the interest of individuals in a group and thereby promotes cooperative self-restraint even in the absence of policing. There are at least two explanations why this might be. First, similarity might have served as a cue for genetic relatedness in the past when self-restraint probably provided indirect benefits due to interactions mostly taking place among related individuals [35]. Although in modern human societies relatedness is actually often low, people might still respond to these cues, irrespective of the adaptive consequences. Second, similarity – although having potentially served as a cue for relatedness in the past – might have turned into a new cue that allows assessing how likely it is to engage in repeated interactions with the same partner and/or how important reputation building is. The idea here is that people in high-similarity societies (i.e. smaller and more homogeneous cantons) exhibit increased cooperative self-restraint because the importance of reciprocal interactions and reputation building increases – two factors that are well known to promote mutualistic cooperation among humans [18], [40]–[42].

The second finding, showing that policing efforts were highest in societies with low similarity indexes, conforms with policing theory because it shows that disproportionally large investments into policing are required to enforce cooperation under conditions where interests among individuals diverge most. These results are in agreement with findings from an experimental study showing that the level of punishment in economic games increases with size of the community the participants originate from [43]. This strongly suggests that humans respond to cues of similarity in their community by adjusting the level of policing and punishment. Moreover, the agreement between my findings and the experimental results from Marlowe et al. [43] nicely illustrates that humans transfer cues from their natural social environments to experimental settings, where they do not necessarily have any implications [44]–[47].

The third finding supports the key prediction of evolutionary policing theory, namely that increased policing efforts reduce crime rates, thereby enforcing cooperation. Investigating the relationship between policing effort and crime rate matches the longstanding interest among economists in finding out whether an increase in the police force can economically be justified because it reduces crime [28]–[32], [48]. This question has led to quite some controversy among the respective researchers in the field because most of the earlier studies revealed that policing efforts in a given year were positively, and not negatively, associated with crime rates [30], [32]. In later studies, it has been recognized that comparisons between the two variables in a given year across geographical entities such as cities and states are confounded by many other factors. Most importantly, data suggested that policing efforts in such analysis were a product of crime rates and not vice versa [30], [32]. To solve that problem, later studies related changes in policing efforts across years [30], [49], [50], electoral cycles [51], and before/after a terrorist attack [52] to changes in crime rates. These comparisons generally revealed that increased policing efforts were indeed associated with lower crime rates. My results are in full agreement with these findings: (a) within-year comparisons revealed positive relationships between policing efforts and crime rates (Table 2, Figure 1C), supporting the previously found reversion of causality; (b) across-year comparisons revealed a negative relationship between the two variables (Table 2, Figure 1D), indicating that increased policing efforts indeed reduce crime rates. In summary, economical and evolutionary approaches both indicate that humans seem to respond to changes in the community policing level by altering their social behaviour. More specifically, policing seems to deter people from committing crimes, thereby enforcing cooperation among citizens.

While I focussed on policing as a mechanism to enforce cooperation, there are a number of other (not necessarily mutually exclusive) mechanisms that have been suggested to also efficiently repress competition among interacting individuals [22], [53]. Among these, costly punishment in humans has certainly received most empirical [12], [43], [54]–[59] and theoretical [60]–[69] attention, with work specifically aiming at identifying factors that facilitate the spread of punishment. For example, it has been shown that costly punishment is more likely favored when it is facultative [61], [68], coordinated at the group level [68], when consequences for defectors are more severe [70], or when cooperation per se is facultative [65], [66]. Furthermore, costly punishment seems to evolve more successfully when acting in concert with other factors known to favor cooperation, such as indirect reciprocity [64] and reputation building [69]. In addition to punishment, rewarding has recently been found to also successfully enforce cooperation among humans [71]–[74]. Along with the policing studied here, these data suggest that multiple repression-of-competition mechanisms might have jointly played a role in the evolutionary maintenance of human cooperative behavior.

Important to note is also that in most laboratory studies the decision whether to punish/reward or not was based on individual choices. This differs from institutional-based enforcement systems, such as the policing institutions analyzed in this study. While both systems seem to be relevant in humans, the question whether individual-based or institutional-based enforcement systems are more successful in promoting cooperation is currently a matter of debate [75], [76].

In conclusion, the analyses presented here indicate that evolutionary policing theory holds for organisms as diverse as humans and social insects – the two groups of organisms, in which sophisticated forms of policing have evolved. Despite this support for evolutionary policing theory, care must be taken not to over interpret the results from comparative approaches used here and in other studies. This is because comparative approaches are based on correlational analysis, which preclude making firm conclusions on the causalities between correlating variables. Hence, a more rigorous test of evolutionary policing theory with humans could be performed in laboratory settings, in which the propensity to cooperate and police could be measured as a function of an experimentally manipulated similarity index.

## References

[1] Darwin CR (1859). The Origin of Species..

[2] Hamilton WD (1964). The genetical evolution of social behaviour.. J Theor Biol.

[3] Lehmann L, Keller L (2006). The evolution of cooperation and altruism - a general framework and a classification of models.. J Evol Biol.

[4] West SA, Griffin AS, Gardner A (2007). Social semantics: altruism, cooperation, mutualism, strong reciprocity and group selection.. J Evol Biol.

[5] Gardner A, West SA, Wild G (2011). The genetical theory of kin selection.. J Evol Biol.

[6] Ratnieks FLW, Foster KR, Wenseleers T (2006). Conflict resolution in insect societies.. Annu Rev Entomol.

[7] Ratnieks FLW (1988). Reproductive harmony via mutual policing by workers in eusocial Hymenoptera.. Am Nat.

[8] Hammond RL, Keller L (2004). Conflict over male parentage in social insects.. PLoS Biol.

[9] Wenseleers T, Ratnieks FLW (2006). Enforced altruism in insect societies.. Nature.

[10] Wenseleers T, Ratnieks FLW (2006). Comparitive analysis of worker reproduction and policing in eusocial Hymenoptera supports relatedness theory.. Am Nat.

[11] Clutton-Brock TH, Parker GA (1995). Punishment in animal societies.. Nature.

[12] Fehr E, Gächter S (2000). Cooperation and punishment in the public goods experiments.. Am Econ Rev.

[13] Kiers ET, Rousseau RA, West SA, Denison RF (2003). Host sanctions and the legume-rhizobium mutualism.. Nature.

[14] Jandér KC, Herre EA (2010). Host sanctions and pollinator cheating in the fig tree–fig wasp mutualism.. Proc R Soc Lond B.

[15] Kümmerli R, van den Berg P, Griffin AS, West SA, Gardner A (2010). Repression of competition promotes cooperation: experimental evidence from bacteria.. J Evol Biol.

[16] Leigh EG (1977). How does selection reconcile individual advantage with the good of the group?. Proc Natl Acad Sci USA.

[17] Alexander RD (1979). Darwinism and Human Affairs..

[18] Alexander RD (1987). The Biology of Moral Systems..

[19] Maynard Smith J, Szathmary E (1995). The major transitions in evolution..

[20] Frank SA (1995). Mutual policing and repression of competition in the evolution of cooperative groups.. Nature.

[21] Frank SA (2003). Perspective: repression of competition and the evolution of cooperation.. Evolution.

[22] West SA, Griffin AS, Gardner A (2007). Evolutionary explanations for cooperation.. Curr Biol.

[23] Gardner A, Grafen A (2009). Capturing the superorganism: a formal theory of group adaptation.. J Evol Biol.

[24] Ratnieks FLW, Wenseleers T (2005). Policing insect societies.. Science.

[25] Fehr E, Fischbacher U (2003). The nature of human altruism.. Nature.

[26] Sigmund K (2007). Punish or perish? Retaliation and collaboration among humans.. Trends Ecol Evol.

[27] Frank SA, Levin SA (2009). Evolutionary foundations of cooperation and group cohesion.. Games, groups, and the global good.

[28] Becker GS (1968). Crime and punishment: an economic approach.. J Polit Econ.

[29] Cameron S (1988). The economics of crime deterrence: a survey of theory and evidence.. Kyklos.

[30] Marvell TB, Moody CE (1996). Specification problems, police levels, and crime rates.. Criminology.

[31] Kaplow L, Shavell S, Auerbach AJ, Feldstein M (2002). Economic analysis of law.. Handbook of Public Economics.

[32] Levitt SD, Miles TJ (2006). Economic contributions to the understanding of crime.. Annu Rev Law Soc Sci.

[33] El Mouden C, West SA, Gardner A (2010). The enforcement of cooperation by policing.. Evolution.

[34] Ledyard JO, Kagel JH, Roth AE (1995). Public good: A survey of experimental research.. The Handbook of Experimental Economics.

[35] Bowles S (2006). Group competition, reproductive leveling, and the evolution of human altruism.. Science.

[36] Efferson C, Lalive R, Fehr E (2008). The coevolution of cultural groups and ingroup favoritism.. Science.

[37] Fischer I (2009). Friend or foe: subjective expected relative similarity as a determinant of cooperation.. J Exp Psychol Gen.

[38] Cornelis I, van Hiel A, de Cremer D (2010). Birds of a feather: Leader-follower similarity and procedural fairness effects on cooperation.. Eur J Work Organ Psychol.

[39] Benjamini Y, Hochberg Y (1995). Controlling the false discovery rate: a pratical and powerful approach to multiple testing.. J R Stat Soc A.

[40] Trivers RL (1971). The evolution of reciprocal altruism.. Q Rev Biol.

[41] Nowak MA, Sigmund K (1998). Evolution of indirect reciprocity by image scoring.. Nature.

[42] Milinski M, Semmann D, Krambeck HJ (2002). Reputation helps solve the ‘tragedy of the commons’.. Nature.

[43] Marlowe FW, Berbesque JC, Barr A, Barrett C, Bolyanatz A (2008). More ‘altruistic’ punishment in larger societies.. Proc R Soc Lond B.

[44] Bateson M, Nettle D, Roberts G (2006). Cues of being watched enhance cooperation in a real-world setting.. Biol Lett.

[45] Haley KJ, Fessler DMT (2005). Nobody's watching? Subtle cues affect generosity in an anonymous economic game.. Evol Hum Behav.

[46] Kümmerli R, Colliard C, Fiechter N, Petitpierre B, Russier F (2007). Human cooperation in social dilemmas: comparing the Snowdrift game with the Prisoner's Dilemma.. Proc R Soc Lond B.

[47] Kümmerli R, Burton-Chellew MN, Ross-Gillespie A, West SA (2010). Resistance to extreme strategies, rather than prosocial preferences, can explain human cooperation in public goods games.. Proc Natl Acad Sci USA.

[48] Ruddell R, Thomas MO (2009). Does politics matter? cross-national correlates of police strength.. Policing.

[49] Corman H, Mocan HN (2000). A time-series analysis of crime, deterrence, and drug abuse in New York City.. Am Econ Rev.

[50] Rosenfeld R, Fornango R, Rengifo AF (2007). The impact of order-maintenance policing on New York City homicide and robbery rates.. Criminology.

[51] Levitt SD (1997). Using electoral cycles in police hiring to estimate the effect of police on crime.. Am Econ Rev.

[52] Di Tella R, Schargrodsky E (2004). Do police reduce crime? Estimates using the allocation of police forces after a terrorist attack.. Am Econ Rev.

[53] Leimar O, Hammerstein P (2010). Cooperation for direct fitness benefits.. Phil Trans R Soc Lond B.

[54] Fehr E, Gächter S (2002). Altruistic punishment in humans.. Nature.

[55] Henrich J, McElreath R, Barr A, Ensminger J, Barret C (2006). Costly punishment across human societies.. Science.

[56] Gürerk O, Irlenbusch B, Rockenbach B (2006). The competitive advantage of sanctioning institutions.. Science.

[57] Gächter S, Herrmann B (2009). Reciprocity, culture and human cooperation: previous insights and a new cross-cultural experiment.. Phil Trans R Soc Lond B.

[58] Egas M, Riedl A (2008). The economics of altruistic punishment and the maintenance of cooperation.. Proc R Soc Lond B.

[59] Wu J-J, Zhang B-Y, Zhou Z-X, He Q-Q, Zheng X-D (2009). Costly punishment does not always increase cooperation.. Proc Natl Acad Sci USA.

[60] Boyd R, Richerson PJ (1992). Punishment allows the evolution of cooperation (or anything else) in sizable groups.. Ethol Sociobiol.

[61] Gardner A, West SA (2004). Cooperation and punishment, especially in humans.. Am Nat.

[62] Lehmann L, Rousset F, Roze D, Keller L (2007). Strong reciprocity or strong ferocity? A population genetic view of the evolution of altruistic punishment.. Am Nat.

[63] Boyd R, Gintis H, Bowles S, Richerson PJ (2003). The evolution of altruistic punishment.. Proc Natl Acad Sci USA.

[64] Rockenbach B, Milinski M (2006). The efficient interaction of indirect reciprocity and costly punishment.. Nature.

[65] Brandt H, Hauert C, Sigmund K (2006). Punishing and abstaining for public goods.. Proc Natl Acad Sci USA.

[66] Hauert C, Traulsen A, Brandt H, Nowak MA, Sigmund K (2007). Via freedom to coercion: the emergence of costly punishment.. Science.

[67] Helbing D, Szolnoki A, Perc M, Szabo G (2010). Evolutionary establishment of moral and double moral standards through spatial interactions.. PLoS Comput Biol.

[68] Boyd R, Gintis H, Bowles S (2010). Coordinated punishment of defectors sustains cooperation and can proliferate when rare.. Science.

[69] dos Santos M, Rankin DJ, Wedekind C (2011). The evolution of punishment through reputation.. Proc R Soc Lond B.

[70] Helbing D, Szolnoki A, Perc M, Szabo G (2010). Punish, but not too hard: how costly punishment spreads in the spatial public goods game.. New J Phys.

[71] Sefton M, Shupp R, Walker JM (2007). The effect of rewards and sanctions in provision of public goods.. Econ Inquiry.

[72] Rand DG, Dreber A, Ellingsen T, Fudenberg D, Nowak MA (2009). Positive interactions promote public cooperation.. Science.

[73] Hilbe C, Sigmund K (2010). Incentives and opportunism: from the carrot to the stick.. Proc R Soc Lond B.

[74] Szolnoki A, Perc M (2010). Reward and cooperation in the spatial public goods game.. Eur Phys J.

[75] Sigmund K, De Silva H, Traulsen A, Hauert C (2010). Social learning promotes institutions for governing the commons.. Nature.

[76] Szolnoki A, Szabo G, Perc M (2011). Phase diagrams for the spatial public goods game with pool punishment.. Phys Rev E.

